# Resveratrol Alleviates Cognitive Impairment in Chronic Cerebral Hypoperfusion by Targeting Lingo-1, NgR1, p75, and RhoA/ROCK-2 Pathways

**DOI:** 10.5812/ijpr-158864

**Published:** 2025-05-20

**Authors:** Mohabbat Jamhiri, Fatemeh Safari, Jalil Alizadeh Ghalenoei, Fatemeh Zare Mehrjerdi, Mansoureh Eslami

**Affiliations:** 1 Neuroscience Research Center, School of Medicine, Shahid Beheshti University of Medical Sciences, Tehran, Iran; 2 Department of Physiology, School of Medicine, Shahid Sadoughi University of Medical Sciences and Health Services, Yazd, Iran; 3 Yazd Neuroendocrine Research Center, School of Medicine, Shahid Sadoughi University of Medical Sciences and Health Services, Yazd, Iran; 4 Department of Basic Sciences, School of Allied Medical Sciences, Shahid Beheshti University of Medical Sciences, Tehran, Iran

**Keywords:** Chronic Cerebral Hypoperfusion, Resveratrol, Lingo-1, NgR1, p75

## Abstract

**Background:**

Chronic cerebral hypoperfusion (CCH), a pathophysiological state linked to vascular dementia and cognitive impairment, involves the NgR1/Lingo-1/p75 signaling complex implicated in neurodegenerative processes. Resveratrol (RES), a neuroprotective compound, was investigated for its potential to mitigate CCH-induced cognitive deficits by targeting this pathway.

**Objectives:**

This study examined RES's ability to improve cognitive impairment in CCH by suppressing the NgR1/Lingo-1/p75 complex and downstream RhoA-ROCK2 signaling.

**Methods:**

Rats were divided into five groups: Control, CCH + Ethanol (ETH), CCH, CCH + resveratrol (RES), and RES. Chronic cerebral hypoperfusion was induced via permanent bilateral carotid artery occlusion (2VO). Cognitive function was assessed using the Morris Water Maze (MWM). Hippocampal morphology in CA1, CA3, and dentate gyrus (DG) regions was analyzed via H&E staining. The expression levels of Lingo-1, NgR1, P75, RhoA, and ROCK2 signaling pathway were detected by western blot and quantitative real-time PCR (qRT-PCR).

**Results:**

Chronic cerebral hypoperfusion rats showed elevated protein expression of Lingo-1, p75, RhoA, and ROCK2, though NgR1 remained unchanged. The RES treatment significantly reduced these protein levels. Similarly, mRNA levels of all five targets increased in CCH, but RES notably lowered Lingo-1 and NgR1 expression. The MWM tests revealed RES improved spatial learning and memory deficits in 2VO rats. H&E staining demonstrated RES’s neuroprotective effects, preserving hippocampal neuron integrity.

**Conclusions:**

Resveratrol alleviates CCH-induced cognitive impairment by downregulating the Lingo-1/NgR1/p75 signaling axis and inhibiting RhoA-ROCK2 pathways. These findings highlight RES’s potential as a therapeutic agent for vascular cognitive impairment associated with chronic hypoperfusion.

## 1. Background

Chronic cerebral hypoperfusion (CCH) is a condition characterized by reduced blood flow to the brain, which is recognized as a primary cause of cognitive decline and dementia (1). The lack of regeneration after CCH injury is a major factor in neurological damage in cerebrovascular diseases (2). Proteins derived from myelin, such as Nogo, myelin-associated glycoprotein (MAG), and oligodendrocyte myelin glycoprotein (OMgp), have been shown to inhibit regeneration in vascular cognitive impairment (3). These proteins bind to the ternary complex of p75NTR, NgR, and LINGO-1. The potential role of the NgR1/Lingo-1/p75-signaling complex in cognitive impairment is particularly intriguing, as this complex can impair learning and memory (4). NgR1 expression in developing nervous systems restricts dendrite growth and axonal growth cone following CNS injury (5). LINGO-1 is a negative regulator of neurite/axon outgrowth that may contribute to hippocampal neuron loss in mental disturbances and cognitive decline (6). Activation of the ternary complex of LINGO-1, NgR, and p75NTR initiates Rho signaling pathways to inhibit neuron outgrowth (7). The inhibitory effects on neurons are mediated by RhoA, with RhoA activation correlating with the inability of axon growth (8). Since vascular cognitive impairment poses a threat to the health and life of patients, it is urgent to find a new strategy for the treatment of cerebral small vessel disease. Resveratrol (RES) is a nutraceutical found in berries, nuts, and red wines (9) that has beneficial biological properties such as antioxidant, anti-inflammatory, and neuroprotective activities (10). A study has shown RES to improve spinal cord injury. A study by Wang et al. demonstrated that RES, a polyphenolic antioxidant, can cross the blood-brain barrier and prevent neuronal cell death (11). Resveratrol reduced neurological dysfunction, neuronal injury, cerebral infarction, and blood-brain barrier (BBB) permeability in a rat model of focal cerebral ischemia (12).

Previous studies have found that RES improves cognitive impairment and memory dysfunction in animal models of dementia (13). Furthermore, a study found that RES promotes neurite outgrowth and synaptogenesis after injury (14). Some studies have shown that RES pretreatment decreased cerebral ischemic injury and improved neurological function (15).

## 2. Objectives

This study investigates the effects of RES on cognitive impairments caused by CCH, focusing on the NgR1/Lingo-1/p75 complex and its downstream signaling pathways.

## 3. Methods

### 3.1. Animals and Experimental Designs

**Figure 1.**
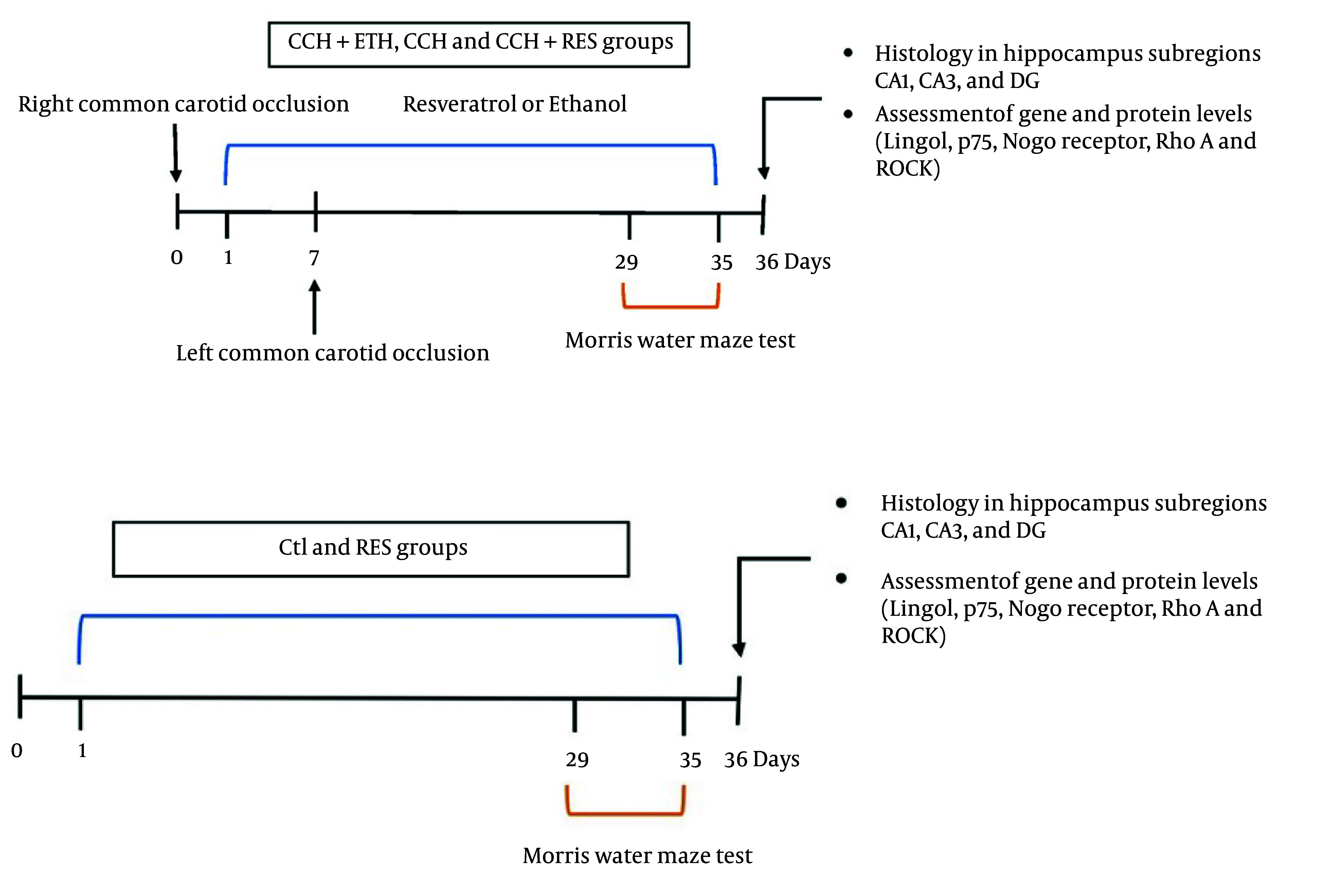
Experimental design–chronic cerebral hypoperfusion (CCH) model was induced by bilateral carotid artery occlusion (2VO). Rats received resveratrol (RES; 5 mg/kg, i.p.) or ethanol daily for 35 days following surgery. Day 0 refers to the day of the surgery.

Sixty male Wistar rats (200 - 230 g) from Shahid Sadoughi University’s Experimental Animal House were housed under controlled conditions (12-hour light/dark cycle, 22 - 25°C) with ethical approval (IR.SBMU.MSP.REC.1400.246). Rats were divided into five groups (n = 12/group) (Figure 1). 

(1) Control: Untreated

(2) CCH + ETH: CCH induced via 2-vessel occlusion (2VO) + ethanol vehicle

(3) The CCH: 2VO-induced CCH only

(4) CCH + Resveratrol (RES): CCH + RES (5 mg/kg/day intraperitoneally for 35 days post-surgery)

(5) The RES: RES alone (no surgery).

Ten of the 2VO rats died during surgery, which were replaced. Resveratrol was dissolved in 0.2% ethanol-distilled water, adhering to prior protocols. Treatments began immediately post-surgery and continued for 35 days (16).

### 3.2. Induction of Bilateral Carotid Artery Occlusion (Chronic Cerebral Hypoperfusion Model)

Animals were anesthetized via intraperitoneal injection of ketamine (40 mg/kg) and xylazine (10 mg/kg). To prevent respiratory distress, atropine (0.1 mg/kg) was administered before surgery. The right common carotid artery was initially occluded by double ligation with silk sutures. One week later, the same procedure was performed on the left common carotid artery. Throughout surgery and recovery from general anesthesia, rectal temperature was maintained at 37.5°C using a heating pad (17).

### 3.3. Behavioral Assessment

#### 3.3.1. Morris Water Maze Test

The MWM test was conducted 28 days after the 2VO surgery to assess the learning and spatial memory of the rats. In each trial, animal behavior was observed for 60 seconds using video tracking, with a maximum of 60 seconds allowed to locate the platform. Over the first four days, the time taken and distance traveled to reach the platform were recorded. On the sixth day, after removing the platform, animals were released into the maze, and their time and distance traveled in the target quadrant were measured to evaluate spatial memory.

### 3.4. Histological Assessment

On day 36, rats were deeply anesthetized with ketamine (70 - 90 mg/kg) and xylazine (10 mg/kg) and then perfused with 200 ml of phosphate-buffered saline (PBS) followed by 200 mL of 4% paraformaldehyde in PBS (pH 7.4). The specimens were cut using a microtome into 5-mm thicknesses for H&E staining. Neuronal necrosis was detected in the hippocampal subregions CA1, CA3, and dentate gyrus (DG).

### 3.5. Western Blot Analysis

Briefly, the hippocampus tissues were homogenized in the RIPA lysis buffer. Protein concentration was measured using the Bradford method at a wavelength of 590 nm. The primary and secondary antibodies used in this study were as follows: β-actin (sc-47778, 1:300), Lingo1 (ab23631, 1:300), NGFR p75 (sc-13577, 1:300), Nogo Receptor (ab184556, 1:300), Rho A (sc-418, 1:300), ROCK2 (sc-398519, 1:300), and secondary antibody (goat anti-rabbit with HRP-conjugated: sc-2357, 1:1000). β-actin was used as an internal loading control for each blot.

### 3.6. RNA Extraction and Real-time RT-PCR Analysis

The hippocampus tissue was lysed with RNX-plus solution and homogenized. First-strand cDNA was synthesized using RevertAid™ M-MuLV Reverse Transcriptase in a total volume of 20 μL. After optimizing the reaction, cDNAs from the experimental groups were obtained using MasterMix containing SYBR green and specific primers in a real-time RT-PCR reaction. Table 1 details the sequence of the primers used in this study. In this research, the beta-actin gene was used as a reference. Relative mRNA expression was calculated by determining the ratio of the target gene expression to the reference using the 2^-ΔΔct^ method.

**Table 1.** Sequence of Primers Used in the Real-time RT-PCR Reaction

Variables	Sequence
**Lingo-1**	
Forward	5′-GGGACAGACAGACGGAAAGC-3′
Reverse	5′-CCGTCTCCTCCTACAACACC-3′
**Nogo receptor (NgR1)**	
Forward	5′-ATGCTACAATGAGCCCAAGG-3
Reverse	5′-GAGCTGTGCATTATCGCTGA-3
**NGFR p75**	
Forward	GTGATGGCAACCTCTACAGT-3′-5′
Reverse	5′-TCTCCACAATGTCAGCTCTC-3′
**Rho A**	
Forward	5′-CGTTAGTCCACGGTCTGGTC-3′
Reverse	5′-CAGCCATTGCTCAGGCAAC-3′
**ROCK**	
Forward	5′-GAAGAGCAGCAGAAGTGGGT-3′
Reverse	5′-GGCAGTTAGCTAGGTTTGTTTGG-3′
**B-actin**	
Forward	5′-CTCTCTTCCAGCCTTCCTTC-3′
Reverse	5′-GGTCTTTACGGATGTCAACG-3′

### 3.7. Statistical Analysis

GraphPad Prism8 was utilized for data analysis. A two-way ANOVA with repeated measures followed by a post-hoc Bonferroni test was employed to analyze the data from the learning test, specifically escape latency and traveled distance. All other data were analyzed using parametric one-way ANOVA followed by Tukey’s post-hoc test to compare differences between groups. All data were expressed as the mean and standard error of the mean (mean ± SEM) (P < 0.05).

## 4. Results

### 4.1. Resveratrol-Alleviated Deficits in Spatial Learning and Memory in Rats Following Chronic Cerebral Hypoperfusion

The results of spatial learning and memory tests in animals showed no significant difference in the distance traveled to reach the platform on the first day. However, on the second day, the CCH and CCH + ETH groups traveled significantly more distance compared to the control group (P < 0.05). The RES group showed a significant reduction in distance traveled compared to the CCH group (P < 0.05).

**Figure 2.**
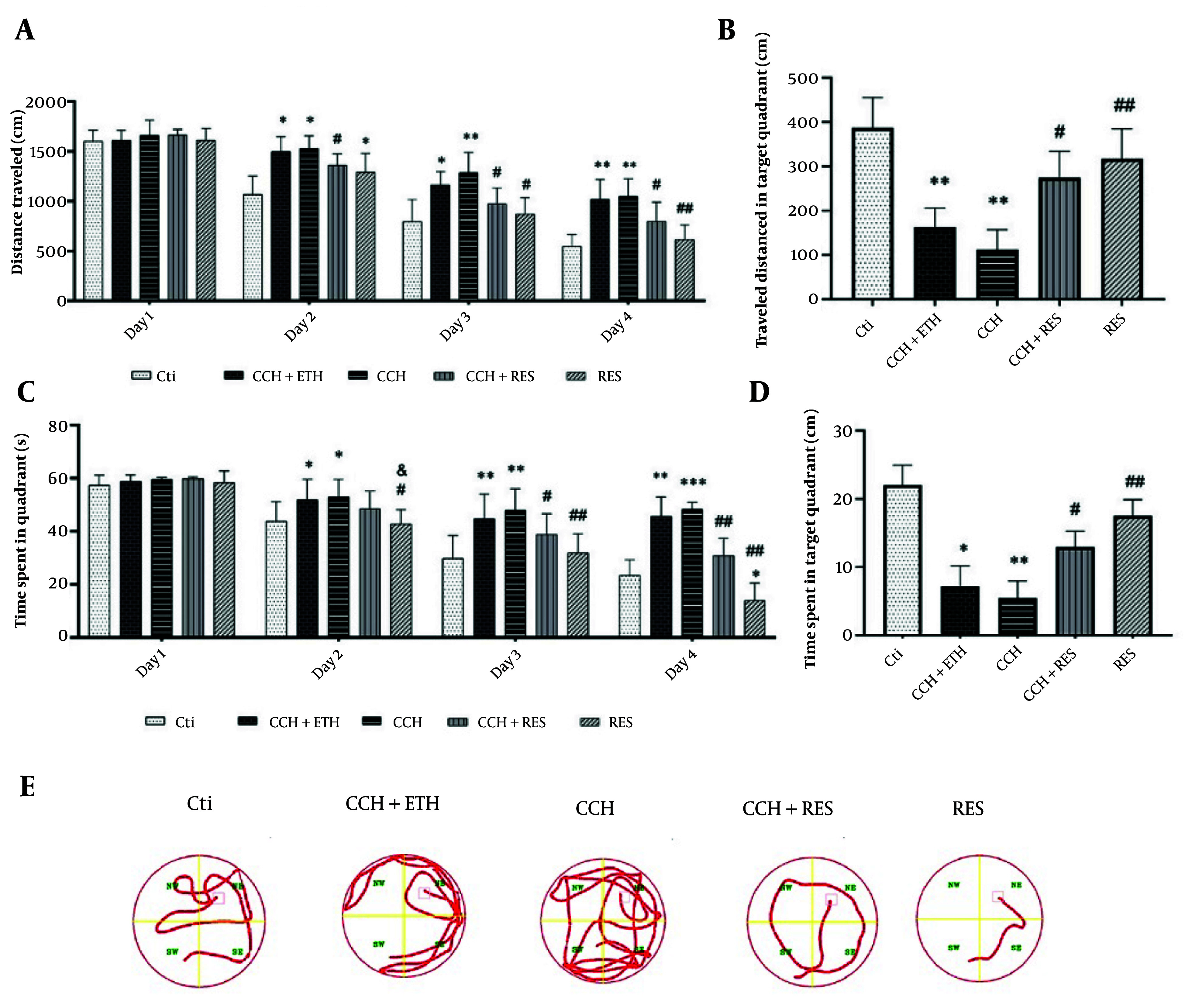
The effects of resveratrol (RES) treatment on spatial learning and memory in rats following chronic cerebral hypoperfusion (CCH) in the Morris Water Maze (MWM) test – A, the distance traveled during the training days; B, the distance traveled in the target quadrant without a platform; C, the time spent during the training days; D, the time spent in the target quadrant without a platform; and E, representative pathways on the last day of training trials. Data are expressed as mean ± SEM (n = 12/group). Differences between groups were determined by ANOVA followed by Tukey's test; * P < 0.05, ** P < 0.01, and *** P < 0.001 vs. Control (Ctl); & P < 0.05 vs. CCH + Ethanol (CCH+ETH); # P < 0.05 and ## P < 0.01 vs. CCH.

On the third day, both CCH and CCH + ETH groups continued to show significant differences in distance traveled compared to the control group (P < 0.05 and P < 0.01, respectively). Additionally, the CCH + RES and RES groups traveled significantly less distance than the CCH group (P < 0.05). On the fourth day, the CCH group still showed a significant difference from the control group (P < 0.05), while the CCH+RES and RES groups traveled significantly less distance than the CCH group (P < 0.05 and P < 0.01, respectively) (Figure 2A). In the target quadrant without a platform, the CCH and CCH + ETH groups showed less activity compared to the CCH group (P < 0.01), while the CCH + RES and RES groups were more active (P < 0.05 and P < 0.01, respectively) (Figure 2B). Regarding the time taken to find the platform, no significant differences were observed on the first day. On the second day, the CCH and CCH + ETH groups took longer than the control group (P < 0.05), while the RES group also showed a significant difference compared to the control (P < 0.05). On the third and fourth days, the CCH group took significantly longer than the control group (P < 0.01 and P < 0.001, respectively). However, the CCH + RES and RES groups showed significant reductions in time compared to the CCH group (P < 0.05 and P < 0.01, respectively) (Figure 2C). The duration spent in the target quadrant was shorter for the CCH group compared to the control (P < 0.01), but longer for the CCH + RES and RES groups compared to the CCH group (P < 0.05 and P < 0.01, respectively) (Figure 2D). Figure 2E demonstrated representative pathways on the last day of training trials.

### 4.2. Resveratrol Treatment Reduced Protein and mRNA Expression Levels of Lingo-1 in Rats Following Chronic Cerebral Hypoperfusion

**Figure 3.**
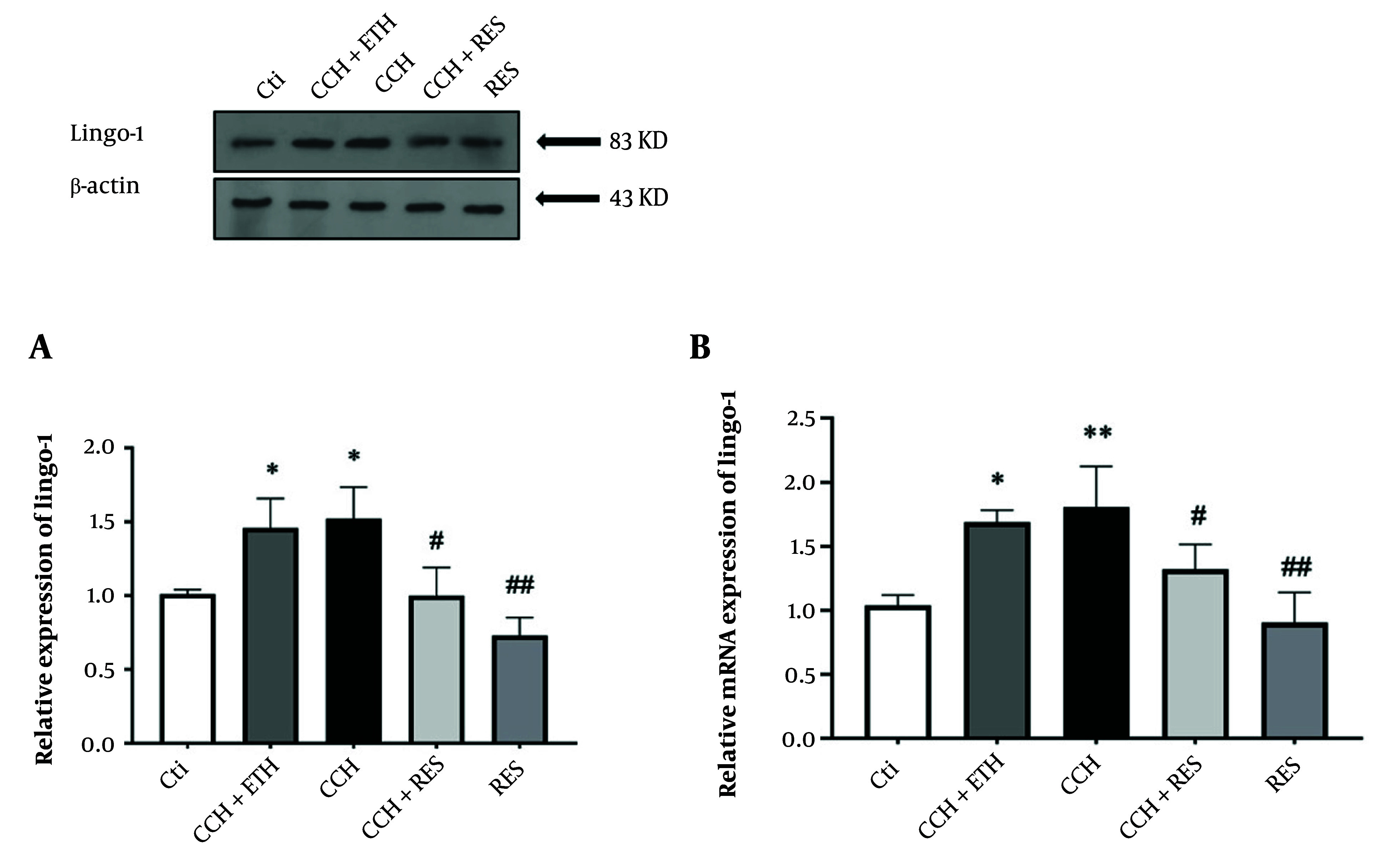
Lingo-1 expression – the protein (A) and mRNA (B) expression levels of Lingo-1 in the hippocampus were assessed in the following groups: Control (Ctl), chronic cerebral hypoperfusion (CCH), CCH + Ethanol (ETH), CCH + Resveratrol (RES) and RES. Results were normalized to β-actin expression. Data are expressed as mean ± SEM (n = 6); * P < 0.05 vs. Ctl, # P < 0.05 and ## P < 0.01 vs. CCH group.

To evaluate the potential mechanisms underlying the neuroprotective effects of RES treatment, the expression levels of Lingo-1, NgR1, P75, RhoA, and ROCK2 were assessed at both mRNA and protein levels. The expression of Lingo-1 protein significantly increased in the CCH + ETH and CCH groups compared to the control group (P < 0.05). However, a decrease in Lingo-1 protein levels was observed in the treatment groups of CCH+RES and RES (P < 0.05) (Figure 3A). Additionally, an increase in Lingo-1 mRNA expression levels was observed following CCH in the CCH + ETH and CCH groups (P < 0.05) compared to the control group. Lingo-1 mRNA levels decreased in the hippocampus after 35 days of RES administration in the CCH+RES and RES groups (P < 0.05 and P < 0.01) (Figure 3B). 

### 4.3. Resveratrol Treatment Reduced Protein and mRNA Expression Levels of NgR1 in Rats Following Chronic Cerebral Hypoperfusion

**Figure 4.**
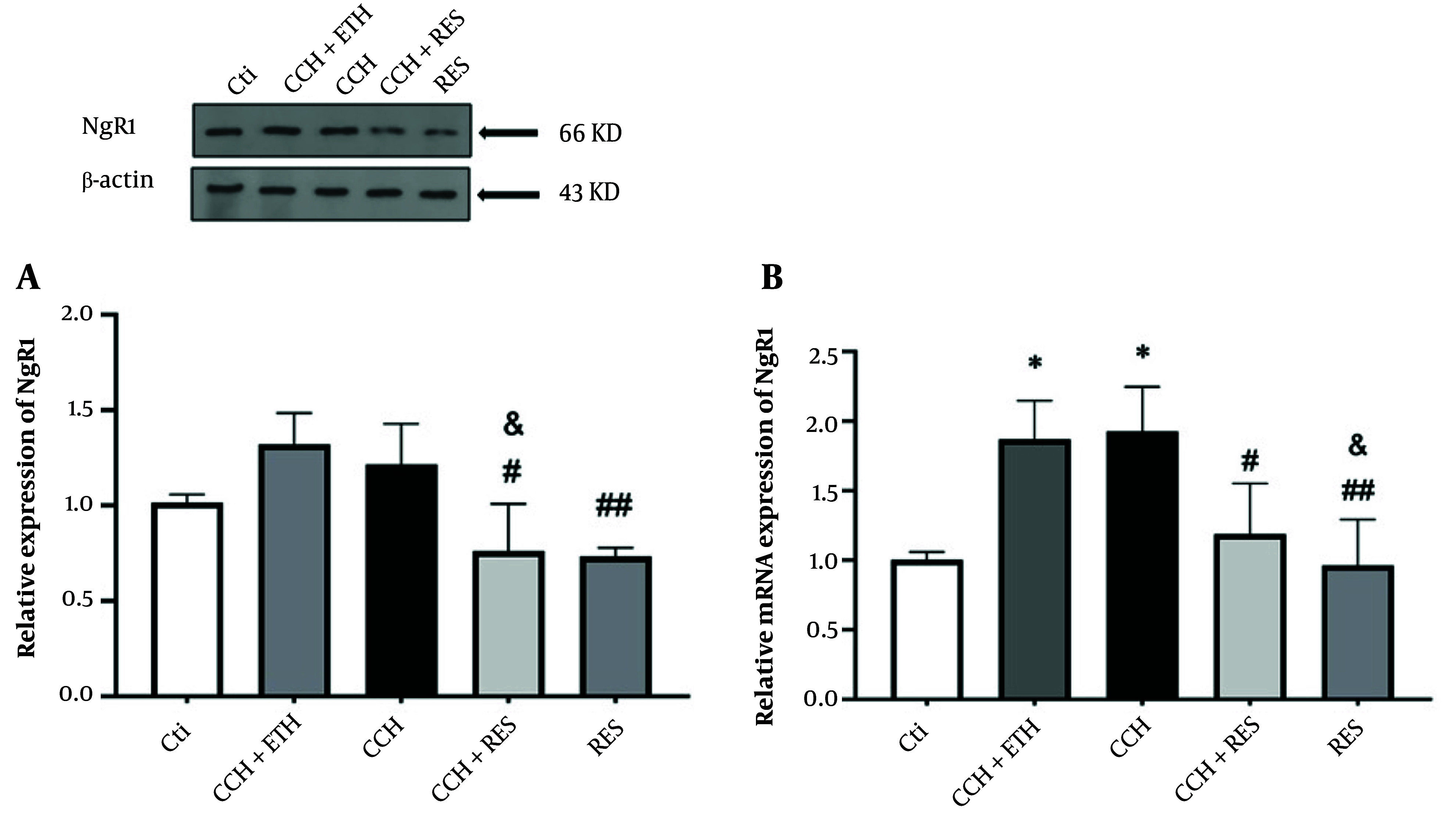
NgR1 expression – the protein (A) and mRNA (B) expression levels of NgR1 in the hippocampus were assessed in the following groups: Control (Ctl), chronic cerebral hypoperfusion (CCH), CCH + Ethanol (CCH + ETH), CCH+Resveratrol (CCH + RES) and resveratrol (RES). Results were normalized to β-actin expression. Data are expressed as mean ± SEM (n = 6); * P < 0.05 vs. Ctl, & P < 0.05 vs. CCH+ETH, # P < 0.05 and ## P < 0.01 vs. CCH group.

Induction of CCH in rats did not show any significant difference in NgR1 protein levels compared to the control groups. However, NgR1 protein expression decreased in the CCH + RES group compared to the CCH+ETH and CCH groups (both at P < 0.05). Additionally, NgR1 levels significantly decreased in the RES group compared to the CCH group (P < 0.01) (Figure 4A). As shown in Figure 4B, mRNA levels of NgR1 in the hippocampus exhibited a significant increase in the CCH and CCH + ETH groups after 35 days (P < 0.05) compared to the control group. NgR1 mRNA levels decreased significantly following CCH in the group treated with RES (P < 0.05). The mRNA expression levels of NgR1 decreased significantly (P < 0.05 and P < 0.01, respectively) in the RES group compared to the CCH + ETH and CCH groups (Figure 4B). 

### 4.4. Resveratrol Treatment Reduced Protein and mRNA Expression Levels of P75 in Rats Following Chronic Cerebral Hypoperfusion

**Figure 5.**
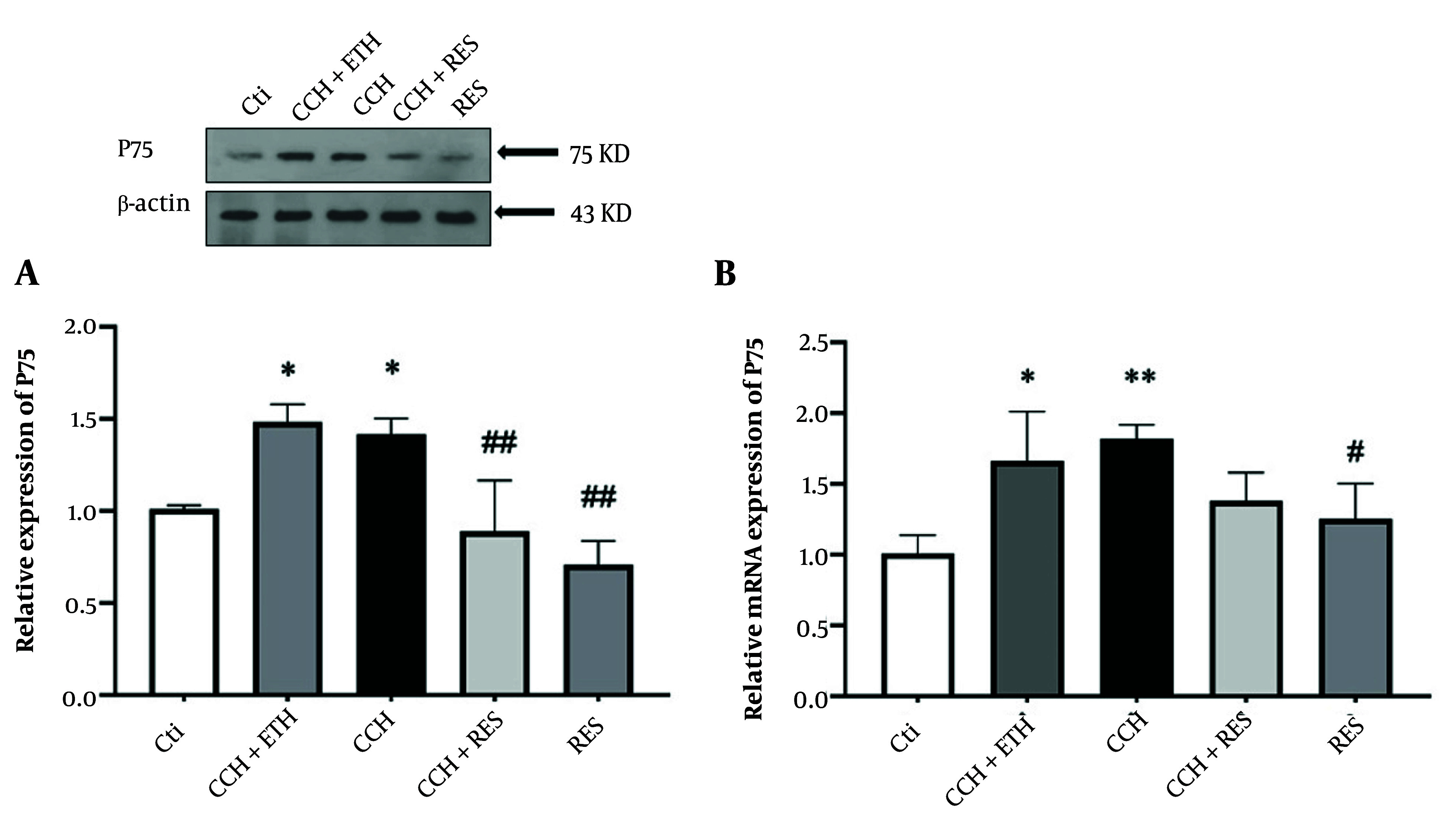
P75 expression – the protein (A) and mRNA (B) expression levels of P75 in the hippocampus were assessed in the following groups: Control (Ctl), chronic cerebral hypoperfusion (CCH), CCH + ethanol (ETH), CCH + resveratrol (RES) and RES. Results were normalized to β-actin expression. Data are expressed as mean ± SEM (n = 6); * P < 0.05 and ** P < 0.01 vs. Ctl, # P < 0.05 and ## P < 0.01 vs. CCH group.

Figure 5A indicated a significant increase in protein levels of P75 in the hippocampus of CCH rats compared to the control groups (P < 0.05). Additionally, P75 protein levels showed a significant reduction in the CCH+RES and RES groups compared to the CCH group (P < 0.01, Figure 5A). P75 mRNA expression levels in hippocampal samples of CCH rats showed a significant increase (P < 0.01) after 35 days compared to the control group (P < 0.05). On the other hand, mRNA expression levels of P75 in the RES group showed a significant reduction (P < 0.05) compared to the control group, while no significant difference was observed in the CCH+RES group compared to the CCH group (Figure 5B). 

### 4.5. Resveratrol Treatment Reduced Protein and mRNA Expression Levels of RhoA in Rats Following Chronic Cerebral Hypoperfusion

**Figure 6.**
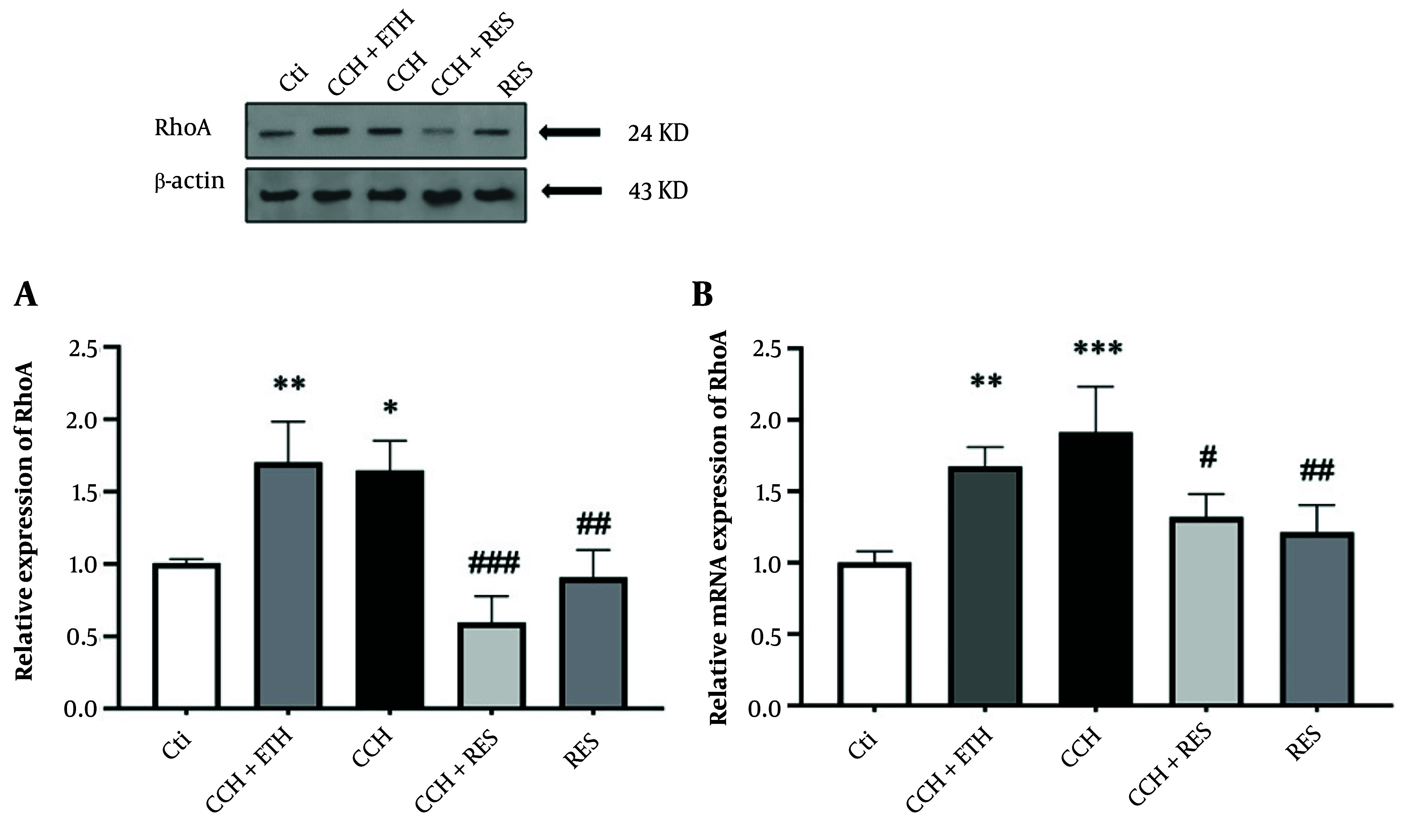
RhoA expression – the protein (A) and mRNA (B) expression levels of RhoA in the hippocampus were assessed in the following groups: Control (Ctl), chronic cerebral hypoperfusion (CCH), CCH + ethanol (ETH), CCH + resveratrol (RES) and RES. Results were normalized to β-actin expression. Data are expressed as mean ± SEM (n = 6); * P < 0.05, ** P < 0.01 and *** P < 0.001 vs. Ctl, # P < 0.05, ## P < 0.01 and ### P < 0.001 vs. CCH.

In the CCH + ETH and CCH groups, the level of RhoA protein showed a significant increase compared to the control (P < 0.05). In the CCH + RES and RES groups, the protein level of RhoA decreased compared to the CCH group (P < 0.001 and P < 0.01, respectively) (Figure 6A). The results of this section also indicated that the expression of mRNA RhoA increased significantly in the CCH and CCH + ETH groups (P < 0.001 vs. control and P < 0.01 vs. control, respectively). However, the mRNA RhoA expression decreased significantly in the CCH+RES group (P < 0.05 vs. CCH). Meanwhile, the mRNA RhoA level showed a significant reduction in the RES group (P < 0.01 vs. CCH) (Figure 6B). 

### 4.6. Resveratrol Treatment Reduced Protein and mRNA Expression Level of ROCK2 in Rats Following Chronic Cerebral Hypoperfusion

**Figure 7.**
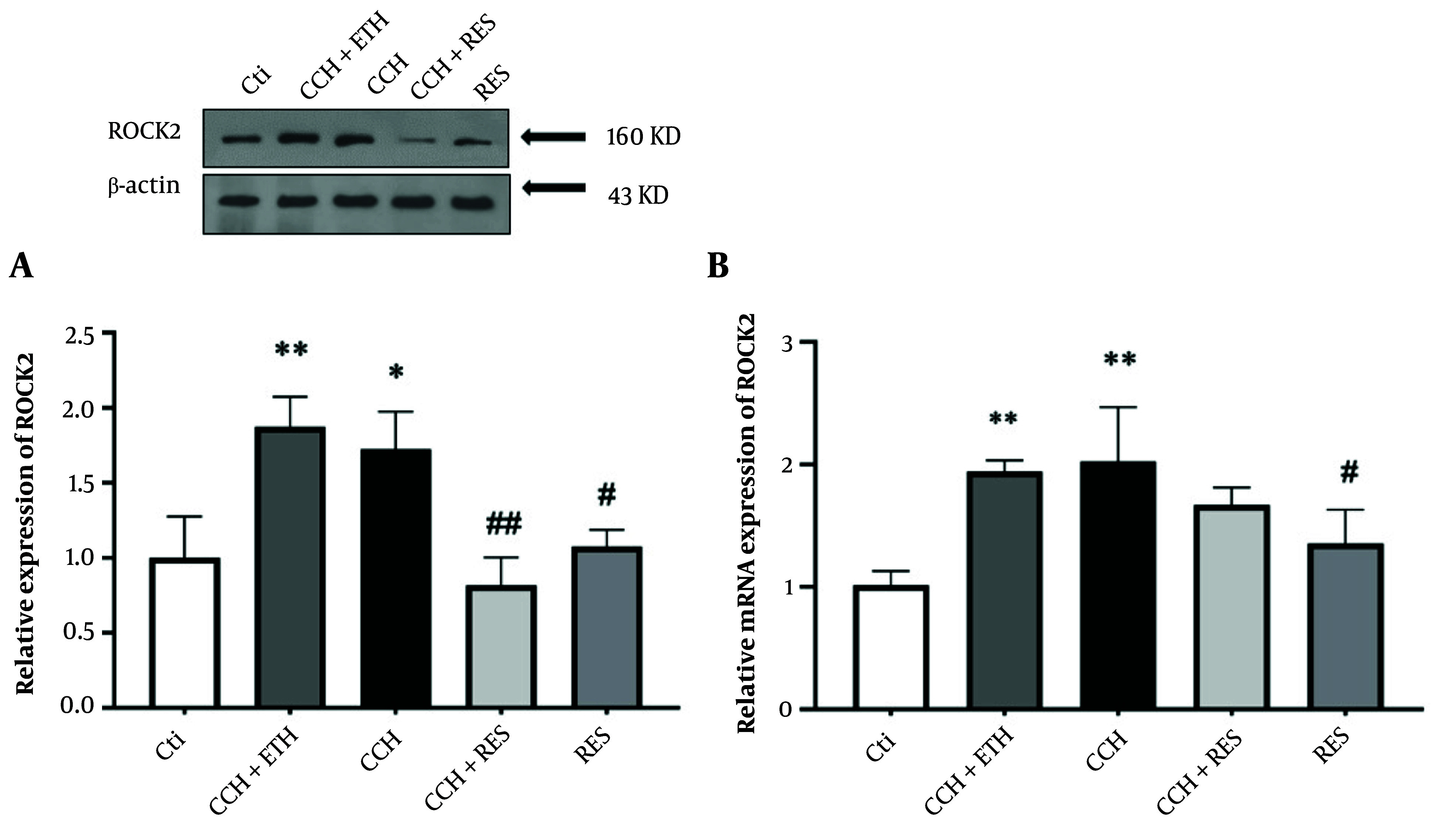
Rock2 expression – the protein (A) and mRNA (B) expression levels of ROCK2 in the hippocampus were assessed in the following groups: Control (Ctl), chronic cerebral hypoperfusion (CCH), CCH + ethanol (ETH), CCH + resveratrol (RES) and RES. Results were normalized to β-actin expression. Data are expressed as mean ± SEM (n = 6); * P < 0.05 and ** P < 0.01 vs. Ctl, # P < 0.05 and ## P < 0.01 vs. CCH.

As shown in Figure 7A, Western blot results indicated that the expression of ROCK2 consistently increased after CCH injury in both the CCH+ETH and CCH groups (P < 0.01 vs. control and P < 0.05 vs. control, respectively). This increase was significantly reversed by RES treatment in the CCH+RES group at 35 days (P < 0.01). Conversely, a decrease in ROCK2 protein level was observed in the hippocampus samples of RES-treated animals without surgical intervention (P < 0.05) compared to the control samples (Figure 7A). Furthermore, a significant increase in the mRNA expression levels of ROCK2 was found 35 days after CCH injury (P < 0.01 vs. control), while RES administration did not show any significant difference in the CCH + RES group compared to the CCH group. The mRNA expression levels of ROCK2 significantly decreased with RES administration (P < 0.05) (Figure 7B). 

### 4.7. Histopathologic Assessment

**Figure 8.**
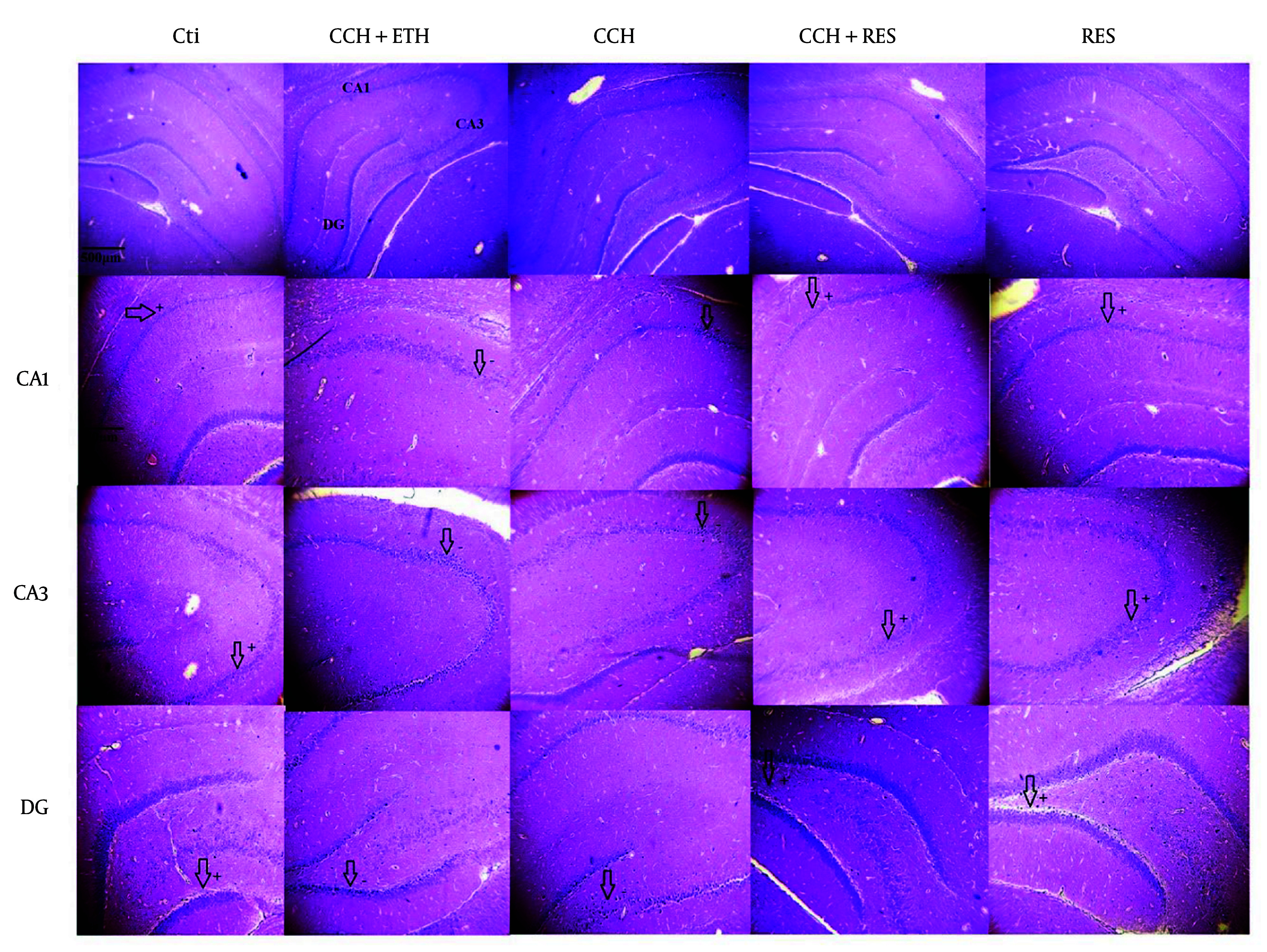
H&E staining in the CA1, CA3 and dentate gyrus (DG) of the rat hippocampus – neuronal death in the hippocampus was assessed in the following groups: Control (Ctl), chronic cerebral hypoperfusion (CCH), CCH + ethanol (ETH), CCH + resveratrol (RES) and RES groups. In the Ctl group, the cytoplasm is stained, and the nuclei are not fragmented in CA1, CA3 and DG. However, neurons are degenerated, and the nuclei are dense in CA1, CA3 and DG in the CCH and CCH + ETH groups. Resveratrol prevented CCH-induced neuronal death in the hippocampus. (Magnification 40X, 400X, scale bar = 200 µm or 50 µm).

Neuronal death following CCH in the hippocampus was examined using H&E staining. Figure 8 shows that the neurons in the CA1, CA3, and DG regions are degenerated with unstained cytoplasm and dense, fragmented nuclei in both the CCH and CCH+ETH groups, indicating cell death in these areas. However, treatment with RES prevented these neuronal changes and reduced cell death in the CA3 and DG regions compared to the CCH group. These results suggest that RES can attenuate CCH-induced neuronal death in the hippocampal subregions CA1, CA3, and DG.

## 5. Discussion

Chronic cerebral hypoperfusion is a common consequence of various cerebral vascular disorders that ultimately lead to cognitive impairment and memory dysfunction (18). In the first part of the current study, we investigated the effect of RES on learning and memory using the MWM test following CCH. The results of the MWM task indicated a significant decrease in spatial learning and memory in CCH rats. However, RES treatment at a dose of 5 mg/kg for 35 days was able to increase the time and distance spent in the target quadrant and reduce the time and distance traveled to find the platform. An important issue to consider in the present study is the possible mechanisms of RES in improving spatial learning and memory impairment. Resveratrol is a non-flavonoid polyphenol that can induce neuroprotection in Alzheimer's disease and other cognitive disorders through modulating the CAMP/AMPK/SIRT1 pathway (19). Zhao et al. revealed that RES treatment improved learning and memory in 8 - 9 month-old mice through the miR-CREB-BDNF pathway (20). Additionally, Li et al. reported that RES treatment enhances the activity of PKA and promotes the phosphorylation of CREB (21). In our study, RES was able to improve learning and memory impairment caused by CCH. We assume that some of the neuroprotective effects of RES are probably due to the inhibition of LINGO-1, NgR1, and p75 protein expression in CCH.

The results of our study showed that the expression levels of Lingo-1, NgR1, and P75 proteins increase following CCH. Additionally, enhanced RhoA-ROCK protein expression was observed in the 2VO rat hippocampus. Zhang and Niu reported results similar to our study in animal models of cerebral ischemia and CCH (22, 23). Several intracellular signaling pathways, such as myelin-associated inhibitory proteins and the NgR1/Lingo-1/p75 complex, play a key role after CCH injury (24). Several studies have shown that LINGO-1, NgR, and p75 expression are increased in spinal cord injury, dementia, Alzheimer's disease, and vascular cognitive impairments (25, 26). Recent studies show that LINGO-1 and NgR, as CNS-specific proteins, are important targets in axonal regeneration and remyelination after CNS injury (3). Wu et al. demonstrated that in the hippocampus of the 5XFAD mice model, the LINGO-1 antibody ameliorates myelin impairment and spatial memory deficits (27). The Lingo-1/NgR/p75 receptor complex exerts its effects via activation of RhoA/ROCK, which mediates the inhibition of neurite growth (7). It has also been reported that myelin-associated inhibitory proteins such as Nogo-66, MAG, and OMgP inhibit axon growth through activation of RhoA, causing degeneration (8). These findings suggest a potential role for the complex in axonal degeneration during cerebrovascular diseases and Alzheimer’s disease. Based on the results of our study and others, it is possible that the observed changes in the expression levels of these proteins are one of the causes of cognitive impairments after CCH injury. Therefore, this complex can be potential targets to promote neural regeneration in neurodegenerative diseases related to vascular cognitive impairment.

In the present study, we report for the first time that treatment with RES for 35 days significantly diminished the mRNA and protein expression levels of Lingo-1, NgR1, and p75 in the CCH rat model. Furthermore, RES significantly reduced the expression of RhoA-ROCK proteins. However, to the best of our knowledge, there is no report on the effect of RES on cognitive impairment induced by CCH through inhibiting the NgR1/Lingo-1/p75 complex. A study has reported that RES improved locomotor and cognitive deficits and modulated SIRT1 and Lingo-1 in harmaline-induced essential tremor (28). Zhou et al. showed that the neuroprotective effects of RES could somehow be exerted through activation of the SIRT1/RhoA signaling pathway (29). Few studies have demonstrated that RES plays a critical role in the pathophysiology of Alzheimer’s disease through the upregulation of SIRT1 and ROCK1 (30). However, the precise mechanisms of RES still have not been fully elucidated in cerebrovascular disease-related cognitive impairment.

Several studies have revealed that some agents, such as roflumilast and RES, enhance cAMP by inhibiting PDEs. The cAMP has been shown to play important roles in neurite outgrowth and neuronal survival. One of the downstream effector molecules of cAMP is Epac, which plays a major role in differentiation, division, and neurotransmission (31). In the present study, it is possible that RES, by inhibiting PDEs and increasing cAMP and Epac, causes the blockage of signal transmission of myelin-associated inhibitors by inducing internalization of NgR1. This suspicion was strengthened through the results of H&E staining in our study.

In the last part, the staining results demonstrated hippocampal CA1, CA3, and DG neuronal degeneration in 2VO rats, while RES treatment decreased the neuronal loss rate in each of the three hippocampal subregions. A study showed that in the CCH rat model, treatment with RES at a dose of 20 mg/kg for 7 days significantly decreased the death of hippocampal CA1 pyramidal neurons and prevented impairments of spatial working memory (32). Studies have shown that the antioxidant properties of RES can play a main role in protection in the hippocampal regions (33, 34). These effects may probably be due to the antiperoxidative property of phenolic compounds in RES. Our results are consistent with previous research that supports RES as a neuroprotective agent in hippocampal neuronal degeneration. However, this study is limited by the lack of histopathological staining to confirm axonal regeneration and synaptogenesis. Future research should focus on elucidating the precise mechanisms of RES in cerebrovascular diseases to identify potential therapeutic targets for cognitive impairments.

### 5.1. Conclusions

This study demonstrates that RES ameliorates neurodegeneration in hippocampal subregions CA1, CA3, and DG following CCH. The neuroprotective effects appear to be mediated primarily through the downregulation of Lingo-1, NgR1, p75, RhoA, and ROCK2 expression. Elucidating the precise mechanisms underlying RES-enhanced axonal regeneration and neuronal survival could reveal novel therapeutic targets for cognitive impairment associated with cerebrovascular disease and vascular dementia.

## Data Availability

The dataset presented in the study is available on request from the corresponding author during submission or after publication.
